# Physical activity and risk of sarcopenia in 6500 community-dwelling Japanese people aged 40–74 years: an 8-year follow-up study

**DOI:** 10.1265/ehpm.25-00046

**Published:** 2025-05-30

**Authors:** Shoya Wakana, Keiko Kabasawa, Kaori Kitamura, Yumi Watanabe, Tomoyo Komata, Yumi Ito, Akemi Takahashi, Toshiko Saito, Ryosaku Kobayashi, Rieko Oshiki, Ribeka Takachi, Shoichiro Tsugane, Kei Watanabe, Junta Tanaka, Ichiei Narita, Kazutoshi Nakamura

**Affiliations:** 1 Division of Preventive Medicine, Niigata University Graduate School of Medical and Dental Sciences, Niigata, Japan; 2 Department of Health Promotion Medicine, Niigata University Graduate School of Medical and Dental Sciences, Niigata, Japan; 3 Department of Rehabilitation, Niigata University of Rehabilitation, Niigata, Japan; 4 Department of Food Science and Nutrition, Nara Women’s University Graduate School of Humanities and Sciences, Nara, Japan; 5 International University of Health and Welfare Graduate School of Public Health, Tokyo, Japan; 6 Department of Orthopedic Surgery, Niigata University Medical and Dental Hospital, Niigata, Japan; 7 Department of General Internal Medicine, Uonuma Kikan Hospital, Niigata, Japan; 8 Division of Clinical Nephrology and Rheumatology, Kidney Research Center, Niigata University Graduate School of Medical and Dental Sciences, Niigata, Japan

**Keywords:** Cohort study, Follow-up survey, Physical activity, Risk factor, Sarcopenia

## Abstract

**Background and aim:**

The association between physical activity (PA) and sarcopenia has mostly been investigated in older people, with few studies focused on earlier life stages. The present study aimed to determine whether higher PA levels are associated with a lower sarcopenia risk in middle-aged and early older people.

**Methods:**

This was an 8-year follow-up study. Participants were 6,500 community-dwelling adults aged 40–74 years who participated in the baseline questionnaire survey conducted in 2011–2014 in Japan. Levels of total and leisure-time PAs at baseline were assessed using validated metabolic equivalent scores. Multi-frequency bioelectrical impedance analysis and handgrip strength measurement were performed in 2021–2022, and participants with low height-adjusted appendicular lean mass (<20th percentile) and low grip strength were diagnosed as having sarcopenia (outcome). Covariates were demographics, body size, lifestyle, and disease history at baseline.

**Results:**

The prevalence of sarcopenia was 137/2926 (4.7%) for men and 127/3574 (3.6%) for women. Higher total PA levels were associated with lower odds of sarcopenia (P for trend = 0.0278), with the second highest group having a significantly lower OR (0.51) than the lowest group (reference) in women, but not in men. Regarding leisure-time PA, those engaged in leisure-time vigorous PA had a lower OR of sarcopenia than those who did not (OR = 0.67, P = 0.0625).

**Conclusion:**

Higher levels of total PA are associated with a lower risk of sarcopenia in women but not in men, suggesting a sex difference in this association. In addition, high levels of vigorous leisure-time PA may be effective for preventing sarcopenia.

**Supplementary information:**

The online version contains supplementary material available at https://doi.org/10.1265/ehpm.25-00046.

## 1. Introduction

Sarcopenia is a progressive and generalized skeletal muscle disorder associated with increased likelihood of adverse outcomes including falls, fractures, physical disability, and mortality [1]. Although the prevalence of sarcopenia varies between studies and depending on definitions used, it has been estimated to range from 10 to 16% in the general older population globally [2]. The economic burden of sarcopenia is also substantial; for example, the direct healthcare costs attributable to sarcopenia in the United States in 2000 were estimated at $18.5 billion, or about 1.5% of total healthcare costs [3].

Physical activity (PA) or exercise is a basic and important factor associated with sarcopenia [4]. Intervention studies demonstrated that physical exercise has protective effects on sarcopenia, including muscle mass and muscle function [5, 6]. However, effects of daily PA on sarcopenia are less clear. Although a number of epidemiologic studies have shown an inverse association between daily PA levels and sarcopenia status, most studies exclusively targeted older people [7, 8]. People in earlier life stages, including middle-aged and early older people, are also important targets for sarcopenia prevention, but only a few studies have been conducted with this population. For example, Zhao et al. [9] reported an inverse association between certain types of physical activity levels and the prevalence of sarcopenia, diagnosed based on appendicular limb muscle mass, in U.S. adults aged 20 to 59 years. Zhao et al. [10] studied 3,760 Chinese adults aged ≥40 years, and reported that frequency, duration, and intensity of PA are inversely associated with the occurrence of sarcopenia.

Daily PA can be classified into the four fundamental domains of household, transportation, work, and leisure time [11, 12], with each having varying health effects. For example, leisure-time PA, especially high-intensity leisure-time PA, has more cardiovascular benefits than other types of PA [12]. Thus, the effects of PA on sarcopenia should be evaluated according to the type and intensity of PA.

We launched two community-based cohort studies on non-communicable chronic diseases in 2011 and have since been following these cohorts [11, 12]. Physical examinations, including body composition and grip strength tests, were performed in some of the cohorts in 2021 and 2022 to identify sarcopenic individuals for the purpose of determining risk factors for sarcopenia. The present study aimed to examine whether higher PA levels are associated with a lower sarcopenia risk in community-dwelling middle-aged and early older people (aged 40–74 years), and whether this association differs depending on the type and intensity of PA.

## 2. Methods

### 2.1 Design and participants

**Fig. 1:**
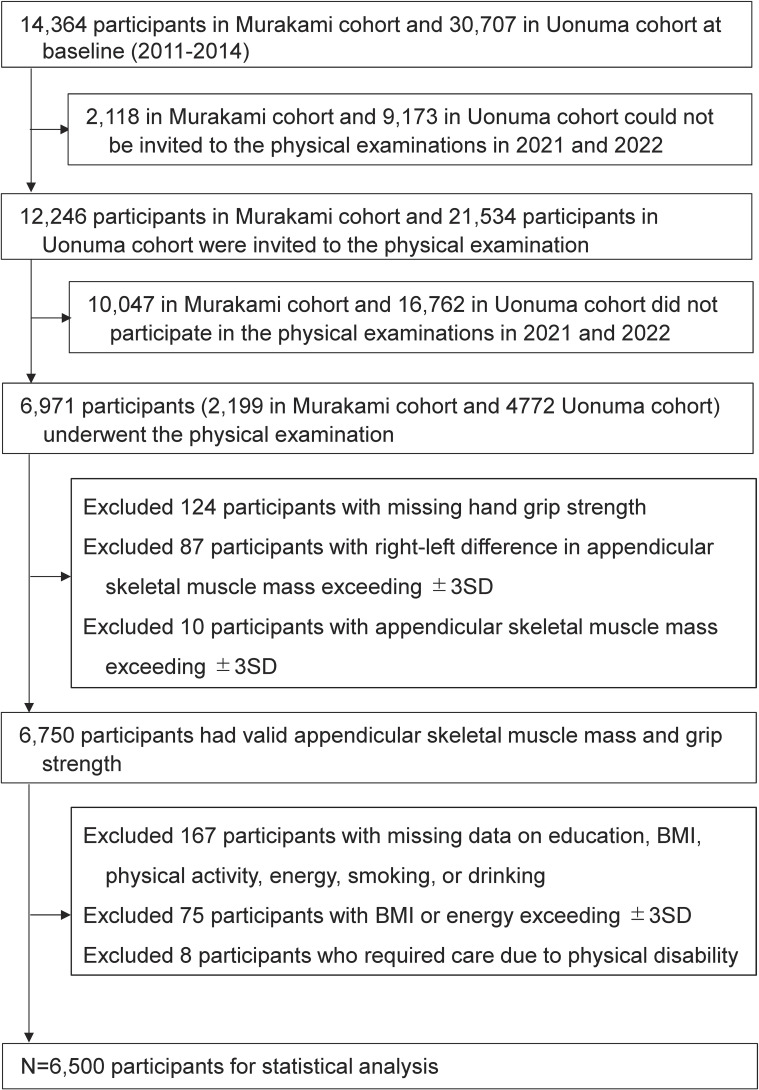
Flow chart of participant selection.

The present study aimed to examine associations between PA levels at baseline in 2011–2014 and sarcopenia status at the follow-up examination conducted in 2021 and 2022 (sarcopenia status was not evaluated at baseline). We used data from a subsample of two population-based cohort studies, the Murakami cohort study (N = 14,364, since 2011) [13] and Uonuma cohort study (N = 39,763, since 2012) [14], in Niigata, Japan. The flow of participant selection is shown in Fig. 1. All participants who could be contacted, i.e., 12,246 in the Murakami cohort and 21,534 in the Uonuma cohort, were invited for the body composition examination by postal mail prior to physical examinations conducted in 2021 and 2022. Of these, a total of 6,971 participants (Murakami, 2,199; Uonuma, 4,772) underwent the examination. Participants with right-left differences in appendicular skeletal muscle mass exceeding ±3SD due to possible hemiplegia or presence of residual metal in the body were excluded. We also excluded eight participants who reported needing care due to a physical disability, as they potentially had sarcopenia at baseline. Ultimately, 6,500 participants were included in the analysis. Written informed consent was obtained from all participants. The study protocol was approved by the Institutional Ethics Committee (Nos. 2019-0376, 2019-0409, and 2022-0240).

### 2.2 Baseline surveys in 2011–2014

The baseline surveys of the Murakami and Uonuma cohort studies were conducted in 2011–2013 and 2012–2014, respectively. Both studies used the same self-reported questionnaire, which elicited information on demographics, body size, lifestyles, and disease histories. Codes for demographic variables, smoking status, and drinking status are shown in Table S1. Levels of PA were assessed with metabolic equivalent (MET) hours per day (MET score) [15], with PA in daily life divided into non-leisure-time PA (commuting, occupational work, and housework, including sitting, standing, walking, and strenuous work) and leisure-time PA (e.g., golf, croquet, and gardening as light to moderate exercise; tennis, jogging, aerobics, and swimming, as strenuous exercise) (Table S1). The questionnaire asked the number of hours spent at each level of intensity for non-leisure-time PA including commuting, occupational work, and housework, and the frequency and number of hours spent at each level of intensity for leisure-time PA, sleep, and other activities. The intensity of each PA is shown in Table S1. To calculate hours spent for other activities, the sum of hours spent for commute, occupational work, housework, leisure-time PA, and sleep was subtracted from 24 hours. This method of measuring PA (the Japan Public Health Center-based prospective study - physical activity questionnaire [JPHC-PAQ]) has been validated previously [15] and was used by our team in other cohort studies on dementia [16] and falls [17]. BMI was calculated as weight (kg) divided by height squared (m^2^). To evaluate general nutritional status, total energy intake was calculated with a validated food frequency questionnaire [18].

### 2.3 PA levels five years later

Five-year questionnaire surveys, conducted in the same manner as the baseline survey, were carried out in both the Murakami and Uonuma cohort studies. Using data from the 5-year survey, we compared PA levels between baseline and five years later.

### 2.4 Physical examination in 2021 and 2022

The body composition examination and hand grip strength measurement were performed during annual medical checkups conducted by the local governments in the Murakami cohort and in the Uonuma cohort in 2021 and 2022. Appendicular lean mass (ALM) was estimated by multi-frequency bioelectrical impedance analysis (MF-BIA) using a standing-posture 8-electrode MF-BIA device (MC-780A-N; TANITA, Tokyo, Japan). The formula for estimating ALM has been published, and a previous validation study demonstrated a high correlation between body composition data obtained using this device and those obtained using dual-energy X-ray absorptiometry [19]. Grip strength was measured twice for each hand using a digital hand dynamometer (Grip-D, TKK-5401; Takei Scientific Instruments Co. Ltd., Niigata, Japan) in the standing position. The highest of the two measurements for each hand was used in the analysis. Sarcopenia was diagnosed with adjusted ALM (ALM/[height]^2^) and grip strength based on criteria of the Asian Working Group for Sarcopenia (AWGS) 2019 [20]; however, the prevalence of sarcopenia determined using the AWGS 2019 criteria was too low, particularly for women [21]. To address this issue, Yamada et al. [21] proposed and validated an alternative cutoff value, the 20th percentile of ALM/[height]^2^, for determining sarcopenia in older populations. We followed this approach and calculated cutoff values of ALM/[height]^2^ to be <7.0 kg/m^2^ for men and <6.1 kg/m^2^ for women. Grip strength cutoffs for sarcopenia were <28 kg for men and <18 kg for women [20].

### 2.5 Statistical methods

Continuous variables were presented as median with interquartile range. P for trend values for participant characteristics were obtained by ordinal logistic regression analysis. Seventy-five extreme values of log-transformed BMI and energy exceeding ±3SD were considered outliers and excluded from the analysis. The outcome was sarcopenia, and primary predictors were total, non-leisure-time, and leisure-time PAs. Logistic regression analysis was used to calculate odds ratios (ORs) for sarcopenia. In multivariate analyses, covariates including marital status, education, occupation, BMI, energy, smoking status, drinking status, and history of stroke and diabetes obtained in the baseline surveys were included in the age-adjusted model. Regarding the coding of discrete variables, education, smoking status, and drinking status were coded as described in Table S1, while marital history and occupation were converted into dummy variables (Table S2). We checked for collinearity; the largest absolute value of Pearson’s correlation coefficients among the covariates was 0.36 for men and 0.38 for women. Model fit indices (Intercept and Covariates) of Akaike Information Criterion (AIC) and Bayesian Information Criterion (BIC) were 2212 and 2226, respectively, for the simple model, and 1928 and 2036, respectively, for the multivariate model of the association between total PA levels and sarcopenia. Logistic regression analyses were also performed using light, moderate, and vigorous PAs as secondary predictors. SAS statistical software (release 9.4, SAS Institute Inc.) was used for all statistical analyses. P < 0.05 was considered statistically significant.

## 3. Results

The mean age of participants at baseline was 62.4 (SD, 7.2) years. The mean follow-up period between the 2011–2014 baseline surveys and the 2021–2022 physical examinations was 9.1 years (SD, 1.6). Participant characteristics according to four levels of total and leisure-time PAs at baseline are shown in Table 1. Participant characteristics at the time of physical examination by sex are shown in Table S3. The prevalence of sarcopenia in the 2021–2022 physical examination was 0/36 (0%) for participants in their 40s, 1/436 (0.2%) for those in their 50s, 26/1856 (1.4%) for those in their 60s, 149/3478 (4.3%) for those in their 70s, and 88/694 (12.7%) for those in their 80s.

**Table 1-1:** Participant characteristics at the baseline survey according to quartiles of total PA levels by sex

**Characteristics**	**Quartiles of total PA (MET-hr/d)**									

	**Men**				P for trend*	**Women**				P for trend*
	
	**Q1 (N = 718)**	**Q2 (N = 734)**	**Q3 (N = 742)**	**Q4 (N = 732)**		**Q1 (N = 866)**	**Q2 (N = 919)**	**Q3 (N = 889)**	**Q4 (N = 900)**	
Age (years)	63 (58,66)	64 (60,69)	64 (60,69)	64 (60,68)	0.0960	63 (57,67)	62 (58,67)	63 (59,67)	63 (59,67)	0.0094
BMI (kg/m^2^)	23.2 (21.3,24.8)	22.9 (21.2,24.6)	22.8 (21.3,24.5)	22.9 (21.2,24.5)	0.3076	22.1 (20.2,24.0)	21.9 (20.0,23.8)	22.0 (20.3,23.9)	22.2 (20.4,24.0)	0.4139
Total PA (MET-h/d)	32.6 (30.9,33.7)	37.9 (36.4,39.7)	45.8 (43.8,48.8)	62.1 (56.2,67.2)	<0.0001	32.9 (31.2,33.9)	36.9 (35.9,38.0)	42.3 (40.5,44.2)	53.9 (49.2,59.8)	<0.0001
Non-leisure-time PA	13.3 (10.3,15.9)	20.0 (17.1,23.2)	29.8 (24.6,34.6)	50.1 (43.2,58.4)	<0.0001	13.3 (10.7,16.3)	20.6 (17.3,23.6)	27.6 (23.6,31.2)	41.6 (35.9,49.8)	<0.0001
Sitting	5.2 (2.6,7.8)	5.2 (2.6,5.2)	2.6 (2.6,5.2)	2.5 (0.7,3.7)	<0.0001	5.2 (2.6,7.8)	5.2 (2.6,7.8)	4.8 (2.6,5.2)	2.6 (2.3,5.2)	<0.0001
Standing or walking	5.5 (2.5,7.0)	10.0 (7.0,14.0)	14.0 (10.0,20.0)	19.1 (13.5,24.9)	<0.0001	5.5 (5.5,9.5)	14.0 (10.0,17.5)	18.0 (14.0,24.0)	22.0 (16.0,28.0)	<0.0001
Strenuous work	0.0 (0.0,3.0)	3.0 (3.0,3.0)	12.0 (12.0,12.0)	28.2 (24.0,36.0)	<0.0001	0.0 (0.0,3.0)	3.0 (0.0,3.0)	3.0 (3.0,12.0)	12.0 (11.3,24.0)	<0.0001
Leisure-time PA	0.3 (0.0,1.5)	1.5 (0.1,4.1)	1.5 (0.0,4.5)	0.6 (0.0,4.3)	<0.0001	0.2 (0.0,1.1)	1.0 (0.0,2.8)	1.6 (0.2,4.5)	1.9 (0.0,7.0)	<0.0001
Energy intake (kcal/d)	2003 (1600,2416)	2110 (1729,2555)	2207 (1797,2678)	2325 (1903,2874)	<0.0001	1778 (1449,2212)	1829 (1489,2258)	1857 (1536,2321)	1971 (1530,2449)	<0.0001
Marital status					0.0065					0.4230
Married	574 (81.3%)	610 (83.6%)	634 (86.4%)	620 (85.3%)		716 (83.1%)	772 (84.5%)	719 (81.4%)	740 (83.3%)	
Never married	85 (12.0%)	70 (9.6%)	61 (8.3%)	67 (9.2%)		27 (3.1%)	28 (3.1%)	24 (2.7%)	12 (1.4%)	
Others^†^	47 (6.7%)	50 (6.8%)	39 (5.3%)	40 (5.5%)		119 (13.8%)	114 (12.5%)	140 (15.9%)	136 (15.3%)	
Education level					<0.0001					0.0242
Junior high school	155 (21.6%)	218 (29.7%)	261 (35.2%)	369 (50.4%)		270 (31.2%)	281 (30.6%)	305 (34.3%)	399 (44.3%)	
High school	375 (52.2%)	363 (49.5%)	345 (46.5%)	284 (38.8%)		403 (46.5%)	421 (45.8%)	388 (43.6%)	389 (43.2%)	
Junior or vocational college	83 (11.6%)	76 (10.4%)	74 (10.0%)	51 (7.0%)		166 (19.2%)	188 (20.5%)	171 (19.2%)	100 (11.1%)	
University graduates	105 (14.6%)	77 (10.5%)	62 (8.4%)	28 (3.8%)		27 (3.1%)	29 (3.2%)	25 (2.8%)	12 (1.3%)	
Occupation					<0.0001					<0.0001
Office/sales/service work	167 (23.5%)	134 (18.5%)	103 (14.1%)	52 (7.3%)		214 (24.8%)	207 (22.6%)	220 (24.8%)	209 (23.4%)	
Manual job	186 (26.1%)	132 (18.2%)	125 (17.1%)	156 (21.8%)		81 (9.4%)	81 (8.9%)	84 (9.5%)	68 (7.6%)	
Professional/management	119 (16.7%)	191 (26.4%)	293 (40.0%)	379 (53.0%)		61 (7.1%)	98 (10.7%)	113 (12.8%)	192 (21.5%)	
Manual	216 (30.3%)	243 (33.6%)	190 (26.0%)	104 (14.5%)		497 (57.6%)	511 (55.8%)	450 (50.8%)	402 (45.0%)	
Others^‡^	24 (3.4%)	24 (3.3%)	21 (2.9%)	24 (3.4%)		10 (1.2%)	18 (2.0%)	19 (2.1%)	23 (2.6%)	
Smoking status					0.4337					0.7831
Non-smoker	135 (18.8%)	161 (21.9%)	143 (19.3%)	153 (20.9%)		750 (86.6%)	801 (87.2%)	774 (87.1%)	793 (88.1%)	
Past smoker	390 (54.3%)	420 (57.2%)	431 (58.1%)	374 (51.1%)		71 (8.2%)	83 (9.0%)	81 (9.1%)	63 (7.0%)	
1–20 cigarettes/day	77 (10.7%)	69 (9.4%)	75 (10.1%)	63 (8.6%)		37 (4.3%)	25 (2.7%)	19 (2.1%)	37 (4.1%)	
≥20 cigarettes/day	116 (16.2%)	84 (11.4%)	93 (12.5%)	142 (19.4%)		8 (0.9%)	10 (1.1%)	15 (1.7%)	7 (0.8%)	
Drinking status					0.7091					0.3917
Non- or rare drinker	128 (17.8%)	132 (18.0%)	119 (16.0%)	141 (19.3%)		511 (59.0%)	547 (59.5%)	532 (59.8%)	549 (61.0%)	
1–149 g ethanol/week	210 (29.2%)	191 (26.0%)	192 (25.9%)	163 (22.3%)		284 (32.8%)	311 (33.8%)	299 (33.6%)	290 (32.2%)	
150–299 g ethanol/week	168 (23.4%)	182 (24.8%)	168 (22.6%)	159 (21.7%)		46 (5.3%)	40 (4.4%)	38 (4.3%)	35 (3.9%)	
300–449 g ethanol/week	112 (15.6%)	130 (17.7%)	162 (21.8%)	142 (19.4%)		15 (1.7%)	16 (1.7%)	15 (1.7%)	21 (2.3%)	
≥450 g ethanol/week	100 (13.9%)	99 (13.5%)	101 (13.6%)	127 (17.3%)		10 (1.2%)	5 (0.5%)	5 (0.6%)	5 (0.6%)	
History of stroke	18 (2.5%)	22 (3.0%)	22 (3.0%)	19 (2.6%)	0.9369	13 (1.5%)	12 (1.3%)	8 (0.9%)	12 (1.3%)	0.5760
History of diabetes	74 (10.3%)	68 (9.3%)	56 (7.5%)	56 (7.7%)	0.0365	33 (3.8%)	39 (4.2%)	31 (3.5%)	38 (4.2%)	0.8740

BMI: body mass index, PA: physical activity, MET: metabolic equivalent

Data are presented as median with interquartile range in parentheses or as a number

*Note*. Cutoff values are 35.2, 41.9, and 52.4 for men’s total physical activity quartiles; 35.0, 39.1, and 46.4 for women’s total physical activity quartiles; 1.1 and 3.8 for men’s leisure-time physical activity tertiles; and 1.1 and 3.6 for women’s leisure-time physical activity tertiles.

*P-for-trend values for discrete variables are calculated for married individuals in marital status, university graduates in education levels, manual workers in occupation, current smokers in smoking status, and drinkers in drinking status.

^†^Divorced, separated, or bereaved

^‡^Security, farming/forestry/fishery, transportation, or labour services

**Table 1-2:** Participant characteristics at the baseline survey according to levels of leisure-time PA by sex

	**Levels of leisure-time PA (MET-hr/day)**									

	**Men**				P for trend*	**Women**				P for trend*
	
	**Zero (N = 955)**	**Tertiles for scores >0**				**Zero (N = 1030)**	**Tertiles for scores >0**			
	
		**Low (N = 640)**	**Medium (N = 658)**	**High (N = 678)**			**Low (N = 840)**	**Medium (N = 855)**	**High (N = 852)**	
Age (years)	62 (58,66)	63 (58,67)	64 (61,69)	66 (63,70)	<0.0001	61 (55,66)	61 (57,66)	63 (59,68)	65 (61,69)	<0.0001
BMI (kg/m^2^)	22.9 (21.0,24.8)	23.1 (21.2,24.6)	23.0 (21.5,24.6)	22.9 (21.3,24.4)	0.4251	22.1 (20.2,24.0)	22.0 (20.1,23.9)	21.9 (20.3,23.9)	22.2 (20.4,24.0)	0.8009
Total PA (MET-h/d)	42.1 (34.0,55.2)	39.4 (33.5,50.7)	39.1 (34.9,46.7)	45.7 (39.0,54.9)	0.0009	37.4 (33.5,44.7)	37.0 (33.6,42.8)	38.5 (35.4,44.5)	44.6 (39.0,51.6)	<0.0001
Non-leisure-time PA	26.7 (16.3,44.7)	23.2 (15.2,38.7)	22.7 (15.6,32.4)	24.6 (18.2,34.7)	<0.0001	23.6 (16.3,34.7)	22.2 (15.6,31.2)	23.0 (16.3,31.6)	25.7 (18.6,34.7)	0.6980
Sitting	2.6 (0.7,5.2)	2.6 (2.5,5.2)	5.0 (2.6,5.2)	2.6 (2.2,5.2)	0.0423	4.3 (2.6,5.7)	5.1 (2.6,7.0)	5.2 (2.6,5.2)	2.6 (2.6,5.2)	0.0211
Standing or walking	13.5 (5.5,20.0)	10.0 (5.5,18.0)	10.0 (5.5,16.2)	12.0 (7.0,18.0)	0.0019	14.0 (9.5,21.7)	14.0 (9.5,20.2)	14.0 (9.5,20.0)	14.0 (10.0,20.0)	0.5559
Strenuous work	12.0 (3.0,24.0)	3.0 (3.0,12.0)	3.0 (3.0,12.0)	10.3 (3.0,12.0)	<0.0001	3.0 (0.0,12.0)	3.0 (0.0,3.0)	3.0 (0.0,8.0)	3.0 (0.0,12.0)	0.4033
Leisure-time PA	0.0 (0.0,0.0)	0.5 (0.3,0.8)	2.1 (1.6,2.7)	7.5 (5.0,11.5)	<0.0001	0.0 (0.0,0.0)	0.4 (0.2,0.7)	2.1 (1.5,2.6)	6.4 (4.5,9.9)	<0.0001
Energy intake (kcal/d)	2135 (1689,2613)	2144 (1756,2607)	2144 (1783,2559)	2194 (1816,2702)	0.0055	1798 (1427,2243)	1800 (1486,2228)	1905 (1541,2328)	1934 (1550,2427)	<0.0001
Marital status					0.0213					0.3065
Married	781 (82.9%)	523 (82.9%)	546 (83.6%)	588 (87.6%)		844 (82.9%)	709 (84.8%)	712 (84.2%)	682 (80.5%)	
Never married	111 (11.8%)	60 (9.5%)	67 (10.3%)	45 (6.7%)		29 (2.8%)	21 (2.5%)	19 (2.2%)	22 (2.6%)	
Others^†^	50 (5.3%)	48 (7.6%)	40 (6.1%)	38 (5.7%)		145 (14.2%)	106 (12.7%)	115 (13.6%)	143 (16.9%)	
Education level					0.0187					0.8206
Junior high school	376 (39.5%)	200 (31.3%)	182 (27.7%)	245 (36.1%)		376 (36.5%)	234 (27.9%)	281 (32.9%)	364 (42.7%)	
High school	432 (45.4%)	302 (47.3%)	325 (49.4%)	308 (45.4%)		456 (44.3%)	414 (49.3%)	379 (44.4%)	352 (41.3%)	
Junior or vocational college	78 (8.2%)	70 (11.0%)	79 (12.0%)	57 (8.4%)		173 (16.8%)	163 (19.4%)	175 (20.5%)	114 (13.4%)	
University graduates	66 (6.9%)	66 (10.3%)	72 (10.9%)	68 (10.0%)		25 (2.4%)	28 (3.3%)	18 (2.1%)	22 (2.6%)	
Occupation					<0.0001					<0.0001
Office/sales/service work	156 (16.8%)	109 (17.3%)	108 (16.5%)	83 (12.4%)		309 (30.2%)	254 (30.4%)	153 (18.0%)	134 (15.8%)	
Manual job	219 (23.6%)	151 (24.0%)	125 (19.1%)	104 (15.5%)		104 (10.2%)	89 (10.6%)	87 (10.2%)	34 (4.0%)	
Professional/management	375 (40.4%)	218 (34.6%)	196 (30.0%)	193 (28.7%)		194 (19.0%)	121 (14.5%)	84 (9.9%)	65 (7.7%)	
Manual	145 (15.6%)	132 (21.0%)	206 (31.5%)	270 (40.2%)		399 (39.0%)	354 (42.3%)	507 (59.6%)	600 (70.8%)	
Others^‡^	33 (3.6%)	20 (3.2%)	18 (2.8%)	22 (3.3%)		17 (1.7%)	18 (2.2%)	20 (2.4%)	15 (1.8%)	
Smoking status					<0.0001					<0.0001
Non-smoker	192 (20.2%)	122 (19.1%)	141 (21.4%)	137 (20.2%)		877 (85.1%)	712 (84.9%)	760 (89.1%)	769 (90.3%)	
Past smoker	448 (47.1%)	360 (56.4%)	383 (58.2%)	424 (62.5%)		84 (8.2%)	91 (10.8%)	61 (7.2%)	62 (7.3%)	
1–20 cigarettes/day	108 (11.3%)	66 (10.3%)	62 (9.4%)	48 (7.1%)		53 (5.1%)	27 (3.2%)	22 (2.6%)	16 (1.9%)	
≥20 cigarettes/day	204 (21.4%)	90 (14.1%)	72 (10.9%)	69 (10.2%)		16 (1.6%)	9 (1.1%)	10 (1.2%)	5 (0.6%)	
Drinking status					0.0524					0.2255
Non- or rare drinker	197 (20.7%)	98 (15.4%)	107 (16.3%)	118 (17.4%)		618 (60.0%)	471 (56.1%)	526 (61.7%)	524 (61.5%)	
1–149 g ethanol/week	225 (23.6%)	174 (27.3%)	187 (28.4%)	170 (25.1%)		324 (31.5%)	299 (35.6%)	274 (32.1%)	287 (33.7%)	
150–299 g ethanol/week	200 (21.0%)	155 (24.3%)	156 (23.7%)	166 (24.5%)		48 (4.7%)	48 (5.7%)	37 (4.3%)	26 (3.1%)	
300–449 g ethanol/week	170 (17.9%)	114 (17.9%)	116 (17.6%)	146 (21.5%)		28 (2.7%)	15 (1.8%)	12 (1.4%)	12 (1.4%)	
≥450 g ethanol/week	160 (16.8%)	97 (15.2%)	92 (14.0%)	78 (11.5%)		12 (1.2%)	6 (0.7%)	4 (0.5%)	3 (0.4%)	
History of stroke	22 (2.3%)	16 (2.5%)	19 (2.9%)	24 (3.5%)	0.1286	17 (1.7%)	8 (1.0%)	8 (0.9%)	12 (1.4%)	0.5208
History of diabetes	73 (7.7%)	56 (8.8%)	57 (8.7%)	68 (10.0%)	0.1134	31 (3.0%)	29 (3.5%)	43 (5.0%)	38 (4.5%)	0.0416

BMI: body mass index, PA: physical activity, MET: metabolic equivalent

Data are presented as median with interquartile range in parentheses or as a number

*Note*. Cutoff values are 35.2, 41.9, and 52.4 for men’s total physical activity quartiles; 35.0, 39.1, and 46.4 for women’s total physical activity quartiles; 1.1 and 3.8 for men’s leisure-time physical activity tertiles; and 1.1 and 3.6 for women’s leisure-time physical activity tertiles.

*P-for-trend values for discrete variables are calculated for married individuals in marital status, university graduates in education levels, manual workers in occupation, current smokers in smoking habits, and drinkers in drinking status.

^†^Divorced, separated, or bereaved

^‡^Security, farming/forestry/fishery, transportation, or labour services

Of the 6,500 participants, 5,851 participated in the 5-year questionnaire survey. Comparison of total PA between the baseline and 5-year surveys by sex are shown in Table S4. Total PA levels decreased on average during the five years regardless of sex. The decline in total PA among men (−1.0 MET-h/d) was significantly larger than that among women (−0.5 MET-h/d), as tested by the Student’s t-test (P = 0.0345). Spearman’s correlation coefficients of total PA levels between the baseline and 5-year surveys were 0.53 for men and 0.51 for women (both P < 0.0001). In addition, 5-year changes in total PA (PA 5 years later minus baseline PA) tended to be smaller in higher quintiles of baseline total PA.

ORs for sarcopenia according to total PA levels at baseline by sex are shown in Table 2. Higher total PA levels were associated with lower odds of sarcopenia (multivariable-adjusted P for trend = 0.0278), with the second highest group (Q3) having a significantly lower OR (multivariable-adjusted OR = 0.51, 95%CI: 0.30–0.88) than the reference group (Q1) in women. No significant associations were observed in men (multivariable-adjusted P for trend = 0.2863) or men and women combined (multivariable-adjusted P for trend = 0.4132).

**Table 2:** Odds ratios (ORs) for sarcopenia according to quartiles of total and non-leisure-time physical activities (PAs)

	**Quartiles of PA (MET-hr/day)**				**P for trend**

	**Q1**	**Q2**	**Q3**	**Q4**	
Total PA					
Men					
Number of cases	27	34	36	40	
Number of controls	691	700	706	692	
Unadjusted OR (95% CI)	1 (ref)	1.24 (0.74–2.08)	1.31 (0.78–2.17)	1.48 (0.90–2.44)	0.1296
Age-adjusted OR (95% CI)	1 (ref)	1.04 (0.62–1.76)	1.03 (0.61–1.73)	1.31 (0.79–2.17)	0.3165
Multivariable-adjusted OR* (95% CI)	1 (ref)	1.08 (0.62–1.86)	1.06 (0.61–1.85)	1.38 (0.78–2.43)	0.2863

Women					
Number of cases	40	39	22	26	
Number of controls	826	880	867	874	
Unadjusted OR (95% CI)	1 (ref)	0.92 (0.58–1.44)	0.52 (0.31–0.89)	0.61 (0.37–1.02)	0.0129
Age-adjusted OR (95% CI)	1 (ref)	0.92 (0.58–1.46)	0.50 (0.29–0.86)	0.59 (0.36–0.99)	0.0081
Multivariable-adjusted OR* (95% CI)	1 (ref)	0.88 (0.55–1.40)	0.51 (0.30–0.88)	0.65 (0.38–1.10)	0.0278

Men and women combined					
Number of cases	67	73	58	66	
Number of controls	1517	1580	1573	1566	
Unadjusted OR (95% CI)	1 (ref)	1.05 (0.75–1.47)	0.84 (0.58–1.20)	0.95 (0.67–1.35)	0.5172
Age-adjusted OR (95% CI)	1 (ref)	0.97 (0.69–1.37)	0.73 (0.51–1.05)	0.89 (0.62–1.26)	0.2604
Multivariable-adjusted OR^†^ (95% CI)	1 (ref)	0.95 (0.67–1.35)	0.74 (0.51–1.08)	0.92 (0.63–1.34)	0.4132

Non-leisure-time PA					
Men					
Number of cases	30	32	31	44	
Number of controls	701	693	700	695	
Unadjusted OR (95% CI)	1 (ref)	1.08 (0.65–1.80)	1.04 (0.62–1.73)	1.48 (0.92–2.38)	0.1227
Age-adjusted OR (95% CI)	1 (ref)	1.07 (0.64–1.79)	1.00 (0.60–1.69)	1.60 (0.98–2.60)	0.0743
Multivariable-adjusted OR* (95% CI)	1 (ref)	1.03 (0.60–1.75)	1.01 (0.59–1.75)	1.71 (1.00–2.92)	0.0639

Women					
Number of cases	45	27	31	24	
Number of controls	815	867	895	870	
Unadjusted OR (95% CI)	1 (ref)	0.56 (0.35–0.92)	0.63 (0.39–1.00)	0.50 (0.30–0.83)	0.0099
Age-adjusted OR (95% CI)	1 (ref)	0.63 (0.38–1.02)	0.72 (0.45–1.16)	0.56 (0.34–0.94)	0.0405
Multivariable-adjusted OR* (95% CI)	1 (ref)	0.65 (0.39–1.07)	0.73 (0.45–1.19)	0.64 (0.37–1.08)	0.1110

Men and women combined					
Number of cases	75	59	62	68	
Number of controls	1516	1560	1595	1565	
Unadjusted OR (95% CI)	1 (ref)	0.76 (0.54–1.08)	0.79 (0.56–1.11)	0.88 (0.63–1.23)	0.4870
Age-adjusted OR (95% CI)	1 (ref)	0.81 (0.57–1.15)	0.84 (0.59–1.19)	0.97 (0.69–1.37)	0.9003
Multivariable-adjusted OR^†^ (95% CI)	1 (ref)	0.80 (0.56–1.15)	0.85 (0.60–1.22)	1.03 (0.72–1.49)	0.8758

*Note*. Cutoff values are 35.2, 41.9, and 52.4 for men’s total PA; 35.0, 39.1, and 46.4 for women’s total PA; 16.1, 24.6, and 38.6 for men’s non-leisure-time PA; and 16.3, 23.6, and 33.2 for women’s non-leisure-time PA.

*Adjusted for age, marital status, education, occupation, BMI at baseline survey, energy, smoking, drinking, and history of stroke and diabetes

^†^Adjusted for sex, age, marital status, education, occupation, BMI at baseline survey, energy, smoking, drinking, and history of stroke and diabetes

ORs for sarcopenia according to non-leisure-time PA levels in the 2011–2014 baseline surveys by sex are also shown in Table 2. Higher non-leisure-time PA levels tended to be associated with lower odds of sarcopenia in women (P for trend = 0.1110); odds of sarcopenia tended to be higher in men (P for trend = 0.0639, marginally significant). No significant associations were observed in men and women combined (multivariable-adjusted P for trend = 0.8758).

ORs for sarcopenia according to quartiles of non-leisure-time PA, including sitting (light PA), standing or walking (moderate PA), and strenuous work (vigorous PA) by sex were calculated. Regarding vigorous PA (Table 3), higher levels were significantly associated with lower odds of sarcopenia (multivariate adjusted P for trend = 0.0052), with the highest quartile having a significantly lower OR (multivariate adjusted OR = 0.48, 95%CI: 0.28–0.82) than the reference group (Q1) in women. This association was not observed in men. There were no significant associations between quartiles of light/moderate PAs and odds of sarcopenia in men or women (Table S5).

**Table 3:** Odds ratios (ORs) for sarcopenia according to quartiles of strenuous work (vigorous non-leisure-time physical activity)

	**Quartiles of strenuous work (MET-hr/day)**				**P for trend**

	**Q1**	**Q2**	**Q3**	**Q4**	
Men					
Number of cases	26	0	71	40	
Number of controls	565	40	1490	694	
Unadjusted OR (95% CI)	1 (Ref)	0	1.04 (0.65–1.64)	1.25 (0.76–2.08)	0.3534
Age-adjusted OR (95% CI)	1 (Ref)	0	1.04 (0.65–1.66)	1.24 (0.74–2.07)	0.3871
Multivariable-adjusted OR* (95% CI)	1 (Ref)	0	1.16 (0.71–1.90)	1.37 (0.78–2.42)	0.2391

Women					
Number of cases	61	3	44	19	
Number of controls	1139	102	1322	884	
Unadjusted OR (95% CI)	1 (Ref)	0.55 (0.17–1.78)	0.62 (0.42–0.92)	0.40 (0.24–0.68)	0.0002
Age-adjusted OR (95% CI)	1 (Ref)	0.55 (0.17–1.80)	0.69 (0.46–1.02)	0.41 (0.24–0.70)	0.0008
Multivariable-adjusted OR* (95% CI)	1 (Ref)	0.59 (0.18–1.96)	0.69 (0.46–1.04)	0.48 (0.28–0.82)	0.0052

*Note*. Because the 25th percentile value for vigorous PA was 0, it was grouped as Q1:0; Q2:>0, 3.0<; Q3:≥3.0, <19.2; and Q4:≥19.2 in men, and as Q1:0; Q2:>0, 3.0<; Q3:≥3.0, <12.0; and Q4:≥12.0 in women.

*Adjusted for age, marital status, education, occupation, BMI at baseline survey, energy, smoking, drinking, and history of stroke and diabetes.

ORs for sarcopenia according to four levels of leisure-time PA by sex are shown in Table S6. Higher levels of leisure-time PA were not significantly associated with lower odds of sarcopenia in men, women, or men and women combined. ORs for sarcopenia according to levels of leisure-time PA by intensity were calculated. Those who engaged in leisure-time vigorous PA had a lower OR of sarcopenia than those who did not in men (multivariable-adjusted OR = 0.56, 95%CI: 0.30–1.04, multivariable-adjusted P = 0.0678) and in men and women combined (multivariable-adjusted OR = 0.67, 95%CI: 0.44–1.02, multivariable-adjusted P = 0.0625) (Table 4). Levels of leisure-time moderate-to-vigorous PA were not significantly associated with the odds of sarcopenia (Table S7).

**Table 4:** Odds ratios (ORs) for sarcopenia according to levels of leisure-time vigorous PA

	**Leisure-time vigorous PA (MET-hr/day)**		**P value**

	**0**	**>0^†^**	
Men			
Number of cases	125	12	
Number of controls	2379	410	
Unadjusted OR (95% CI)	1 (Ref)	0.56 (0.31–1.02)	0.0566
Age-adjusted OR (95% CI)	1 (Ref)	0.53 (0.29–0.98)	0.0420
Multivariable-adjusted OR* (95% CI)	1 (Ref)	0.56 (0.30–1.04)	0.0678

Women			
Number of cases	112	15	
Number of controls	2970	477	
Unadjusted OR (95% CI)	1 (Ref)	0.83 (0.48–1.44)	0.5156
Age-adjusted OR (95% CI)	1 (Ref)	0.73 (0.42–1.26)	0.2553
Multivariable-adjusted OR* (95% CI)	1 (Ref)	0.79 (0.45–1.39)	0.4054

Men and women combined			
Number of cases	237	27	
Number of controls	5349	887	
Unadjusted OR (95% CI)	1 (Ref)	0.69 (0.46–1.03)	0.0688
Age-adjusted OR (95% CI)	1 (Ref)	0.62 (0.41–0.94)	0.0234
Multivariable-adjusted OR* (95% CI)	1 (Ref)	0.67 (0.44–1.02)	0.0625

*Note*. Leisure-time vigorous PA included strenuous exercise.

*Adjusted for sex, age, marital status, education, occupation, BMI at baseline survey, energy, smoking, drinking, history of stroke and diabetes, and non-leisure-time PA.

^†^Participants were grouped as 0 and >0 (median, 2.3 for men and 1.9 for women) because the 75th percentile values for leisure-time vigorous PA were 0.

## 4. Discussion

The present follow-up study yielded the following findings: 1) higher levels of total PA were associated with a lower risk of sarcopenia in women, but not in men; 2) the inverse association between PA and sarcopenia risk in women was robust for vigorous PA; and 3) those who engaged in vigorous leisure-time PA tended to be at lower risk of sarcopenia than those who did not.

Higher levels of total PA were dose-dependently associated with a lower risk of sarcopenia, with an OR of 0.65 (for the highest to lowest quartiles) in women. Meta-analysis studies have provided sufficient evidence for the association between PA levels and the risk of sarcopenia in older people [7, 8]. However, only a few studies on this topic have been conducted in individuals at earlier life stages. Zhao et al. [9] reported that higher levels of total PA are associated with a lower prevalence of sarcopenia in U.S. adults aged 20 to 59 years, and that the inverse association was more robust in men than women. However, that study [9] used only appendicular muscle mass to define sarcopenia, which differs from the standard definition [1, 20], and thus their results may not be comparable to ours. Zhao et al. [10] reported that the frequency, duration, and volume of PA are inversely associated with the occurrence of sarcopenia in Chinese adults aged ≥40 years, although they did not use METs scores and did not evaluate sex-dependent associations. Sipilä et al. [22] showed that high PA levels were associated with high ALM among late perimenopausal and postmenopausal women, and suggested that being physically active is important for women who have already experienced a significant decline in sex hormone production. Therefore, differences in sex hormone status may explain the sex-dependent variations in the association between total PA levels and sarcopenia status observed in the present study.

The inverse association between non-leisure-time PA and sarcopenia risk in women was more robust for vigorous PA than for light and moderate PAs. Mijnarends et al. [23] reported that moderate-to-vigorous PA levels were inversely associated with 5-year occurrence of sarcopenia, which is in line with our findings. The present study adds to the existing knowledge by clarifying that vigorous PA is more beneficial than moderate PA, although this finding was limited to women.

The preventive effect of PA on sarcopenia has several mechanisms. PA, or physical exercise, improves insulin sensitivity and protein anabolic actions of insulin on skeletal muscle [24]. In addition, PA is associated with an increase in SHBG, which contributes to the bioavailability of androgens and estrogens [25], with an anabolic effect to promote muscle protein synthesis and a protective effect on skeletal muscle by attenuating inflammation, respectively [26].

Higher levels of total PA were expected to reduce the risk of sarcopenia in men as well as in women, given that age-related pathological changes in skeletal muscle that lead to muscle wasting have been suggested to be similar between men and women, despite the sex differences in age-related muscle wasting due to respective sex hormones [27]. However, the present study did not detect an association between total PA levels and sarcopenia risk in men. Rather, the highest quartile of total PA and strenuous work appeared to be associated with an even higher risk of sarcopenia (although insignificant) in men. The reason for this is unclear but may be explained by the findings of a study showing that high total PA levels were associated with a high risk of knee osteoarthritis, while no such association was observed in women [28]. This suggests that high levels of PA in daily life or work, e.g., strenuous work, could cause musculoskeletal disorders in older men, who work at a higher intensity than women. In addition, total PA declined the most over five years in the highest baseline total PA quartile group (Table S4), and a greater decline in total PA tended to be associated with sarcopenia (P for trend = 0.1105 in men in Table S8). This suggests that the decline in total PA may partly account for the association between baseline total PA levels and sarcopenia risk, particularly in men, whose decline in total PA was greater than that in women. In fact, when we introduced the variable ‘5-year change in total PA’ as an additional covariate in the multiple logistic regression analysis, the multivariable-adjusted OR for the highest quartile in men decreased from 1.38 (shown in Table 2) to 1.15 (data not shown), although the causes of the decline in PA remained unclear. Collectively, high levels of PA (e.g., strenuous work) may not necessarily be advantageous in terms of preventing muscle wasting, and rather bring about potentially harmful effects. On the other hand, a large-scale cross-sectional study on middle-aged and older adults reported that occupational PA is not associated with muscle mass [29]. Further research on the relationship between total or non-leisure-time PA and sarcopenia is warranted.

In the present study, participants who engaged in vigorous leisure-time PA tended to be at lower risk of sarcopenia (OR = 0.67, 95%CI: 0.44–1.02, P = 0.0625) than those who did not, consistent with many previous reports. Jacob et al. [8] showed that, among 14,585 older adults (≥65 years) who participated in an international cross-sectional study, those with high leisure-time PA (>150 min/week of moderate-to-vigorous LTPA) had a lower OR (0.54, 95%CI: 0.38–0.77) than those with low leisure-time PA (≤150 min/week). In a cross-sectional study conducted by Rosique-Esteban et al. [30] targeting 1,539 older Spanish adults with overweight/obesity and metabolic syndrome (mean age, 65 years), those with higher levels of both moderate and vigorous PAs had significantly lower ORs. Although the present study found no significant association between moderate-to-vigorous leisure-time PA and sarcopenia risk, the observed trend was similar (P for trend = 0.1879 for men and woman combined). A future study with a larger sample size may clarify this association.

The participation rate was as low as 20.6% (6971/33,780) in the present study. Several factors may explain this. For instance, the physical examination was conducted mostly during the daytime on weekdays, which made it difficult for working individuals to participate. Furthermore, there may have been a tendency to avoid long physical examinations.

Because the present study used a subsample of the original cohorts of the Murakami and Uonuma studies, we compared the basic characteristics of the current subsample with the total sample of the two cohorts. Differences in percent frequencies of age, sex, and total PA quartiles at baseline between the current subsample and the total sample of the two cohorts are shown in Table S9. Younger individuals (<60 years), men, and those with low levels of total PA (MET score <35.0) tended to not participate in the physical examinations conducted in 2021–2022. Moreover, individuals with low PA levels may have dropped out due to other underlying diseases, in which case the odds of sarcopenia in the low PA group and the strength of the association between PA levels and sarcopenia risk may have been underestimated. Furthermore, the physical examination was often conducted in conjunction with annual health check examinations offered by the local government. As a result, individuals with higher health consciousness, who are typically more likely to participate in health checkups [31], may have been more inclined to participate, in which case the present findings may not accurately reflect the general population.

The strength of the present study lies in examining the association between type- and intensity-stratified, METs-based PA levels and sarcopenia status within a framework of large cohort studies targeting middle-aged and early older people. However, the present study also has some limitations worth noting. First, sarcopenic status at the baseline survey of sarcopenia cases was unknown, i.e., some participants may have had sarcopenia at the time of the baseline survey. This could have resulted in an overestimation of ORs because past sarcopenic status could influence PA levels, and in a modification of the causality between PA and the occurrence of sarcopenia. These are a major limitation of the present study. Second, PA levels were assessed in a self-reported manner, which may have led to the misclassification of PA levels. Although the MET score (MET-h/d) obtained using our method (JPHC-PAQ) correlated well with that obtained using the 24-hour activity records method (Spearman’s correlation coefficients for total PA: 0.789 for men and 0.579 for women) [13], potential underestimation of the OR may have occurred. Third, bioelectrical impedance analysis is influenced by an individual’s hydration status. This might have affected the assessment of ALM and the diagnosis of sarcopenia, although participants in the present study were generally healthy. Fourth, there may have been other confounders, including genetic factors, that the present study did not address. Finally, participants in the present study were from regions with medium- and small-sized local governments. Therefore, our results may not apply to regions with large local governments or metropolitan governments (e.g., Tokyo) due to differences in lifestyle.

## 5. Conclusions

The present follow-up study provides evidence supporting a sex difference in the association between PA levels and sarcopenia risk in community-dwelling middle-aged and older people. Higher levels of total PA were associated with a lower risk of sarcopenia in women but not in men. Thus, PA interventions should be provided separately for men and women. In addition, high-level vigorous leisure-time PA may offer an effective means to prevent sarcopenia.

## Supplementary information

**Additional file 1:** **Table S1.** Codes for demographic and lifestyle variables. **Table S2.** Codes for marital status and occupation after conversion to dummy variables. **Table S3.** Participant characteristics in the 2021–2022 physical examinations by sex. **Table S4.** Comparison of total physical activity (PA) among participants between the baseline and 5-year surveys according to sex and baseline total PA quartiles. **Table S5.** Odds ratios (ORs) for sarcopenia according to quartiles of non-leisure-time PA during sitting and standing/walking. **Table S6.** Odds ratios (ORs) for sarcopenia according to four levels of leisure-time physical activity (PA). **Table S7.** Odds ratios (ORs) for sarcopenia according to levels of leisure-time moderate-to-vigorous PAs. **Table S8.** Odds ratios (ORs) for sarcopenia according to quartiles of changes in total PA during the five years. **Table S9.** Differences in percent frequencies of age, sex, and total physical activity (PA) quartiles at baseline between the subsample and the total sample of the two cohorts.

## Declarations

### Data availability statement

We cannot provide individual data because study participants did not consent to have their data provided to anyone outside of the research group. However, a minimal dataset may be available upon ethical approval by the Ethics Committee of Niigata University.
