# Supplementary material for: Physical activity and risk of sarcopenia in 6500 community-dwelling Japanese people aged 40–74 years: an 8-year follow-up study
Source: Environ Health Prev Med. 2025 May 30;30:44. doi: 10.1265/ehpm.25-00046 (PMC12127081; doi:10.1265/ehpm.25-00046)
Supplement: Supplementary file 1 — Additional file 1: Table S1. Codes for demographic and lifestyle variables. Table S2. Codes for marital status and occupation after conversion to dummy variables. Table S3. Participant characteristics in the 2021–2022 physical examinations by sex. Table S4. Comparison of total physical activity (PA) among participants between the baseline and 5-year surveys according to sex and baseline total PA quartiles. Table S5. Odds ratios (ORs) for sarcopenia according to quartiles of non-leisure-time PA during sitting and standing/walking. Table S6. Odds ratios (ORs) for sarcopenia according to four levels of leisure-time physical activity (PA). Table S7. Odds ratios (ORs) for sarcopenia according to levels of leisure-time moderate-to-vigorous PAs. Table S8. Odds ratios (ORs) for sarcopenia according to quartiles of changes in total PA during the five years. Table S9. Differences in percent frequencies of age, sex, and total physical activity (PA) quartiles at baseline between the subsample and the total sample of the two cohorts. [file ehpm-30-044-s001.docx]

Table S1. Codes for demographic and lifestyle variables

Variables

Marital status (a) married, (b) never married, and (c) divorced, separated, or bereaved

Education level (1) junior high school, (2) high school, (3) junior or vocational college, and (4) university graduates

Occupation (a) office work and sales/service work, (b) professional/management, (c) manual (security, farming/forestry/fishery, transportation, and labour services), and (d) no job/housewife and others

Smoking (1) non-smoker, (2) past smoker, (3) 1-20 cigarettes/day, and (4) ≥20 cigarettes/day

Drinking (1) non- or rare drinker, (2) 1-149, (3) 150-299, (4) 300-449, and (5) ≥450 g ethanol/week

Intensity of PA Non-leisure-time PA including commuting, occupational work, and housework (light PA: sitting [1.3 METs],

moderate PA: standing [2.0 METs] and walking [3.0 METs]; and vigorous PA: strenuous work [6.0 METs])

Leisure-time PA (walking slowly [2.8 METs], walking quickly [4.0 METs], light to moderate exercise

[3.0 METs], and strenuous exercise [6.0 METs])

Sleep (0.9 METs)

Other activities (1.3 METs)

Table S2. Codes for marital status and occupation after conversion to dummy variables

Variables

Marital status Never married: (0) No and (1) Yes

Divorced, separated, or bereaved: (0) No and (1) Yes

Occupation Office work or sales/service work: (0) No and (1) Yes

Professional/management: (0) No and (1) Yes

Manual worker (security, farming/forestry/fishery, transportation, and labour services): (0) No and (1) Yes

Table S3. Participant characteristics in the 2021–2022 physical examinations by sex

Men (N=2926) Women (N=3574)

Appendicular lean mass (ALM) (kg) 20.5 (18.2,22.8) 14.9 (13.6,16.2)

Adjusted ALM by height square (kg/m^2^) 7.7 (7.1,8.2) 6.5 (6.2,6.8)

Grip strength (kg) 37.3 (33.0,41.5) 24.3 (21.7,26.9)

Number of low adjusted ALM^*^ 579 (19.8%) 706 (19.8%)

Number of low grip strength^†^ 211 (7.2%) 202 (5.7%)

Number of sarcopenia 137 (4.7%) 127 (3.6%)

*Note*: Median with inter-quartile range in parentheses or percent

^*^Cutoff values of adjusted ALM are <7.0 kg/m^2^ for men and <6.1 kg/m^2^ for women.

^†^Cutoff values of grip strength are <28 kg for men and <18 kg for women.

Table S4. Comparison of total physical activity (PA) among participants between the baseline and 5-year surveys according to sex and baseline total PA quartiles

Total PA levels (MET-h/d)

Baseline 5 Year later Change^*^ Spearman’s correlation

coefficient^†^

Men (N=2649) 44.9 (12.0) 43.9 (10.9) −1.0^‡^ (11.1) 0.53

Quartile 1 (N=642) 32.4 (1.9) 37.1 (7.3) 4.8 (7.5)

Quartile 2 (N=674) 38.1 (1.9) 40.5 (8.1) 2.4 (8.2)

Quartile 3 (N=672) 46.4 (3.0) 45.5 (9.6) −0.9 (9.6)

Quartile 4 (N=661) 62.5 (6.9) 52.1 (11.9) −10.3 (12.1)

Women (N=3202) 41.8 (9.5) 41.4 (9.0) −0.5 (9.1) 0.51

Quartile 1 (N=781) 32.5 (1.7) 36.3 (5.8) 3.7 (5.8)

Quartile 2 (N=831) 36.9 (1.2) 39.1 (6.7) 2.1 (6.7)

Quartile 3 (N=792) 42.4 (2.1) 42.0 (7.9) −0.5 (7.8)

Quartile 4 (N=798) 55.5 (7.5) 48.2 (10.3) −7.3 (11.0)

Overall (N=5851) 43.2 (10.8) 42.5 (10.0) −0.7 (10.1) 0.52

Quartile 1 (N=1423) 32.5 (1.8) 36.7 (6.5) 4.2 (6.7)

Quartile 2 (N=1505) 37.5 (1.7) 39.7 (7.4) 2.3 (7.4)

Quartile 3 (N=1464) 44.2 (3.2) 43.6 (8.9) −0.6 (8.7)

Quartile 4 (N=1459) 58.6 (8.0) 50.0 (11.2) −8.7 (11.6)

*Note*: Mean with SD in parentheses; Quartiles are based on baseline total PA

^*^PA 5 years later minus PA at baseline. All P values are <0.0001, as tested by the paired t-test.

^†^Correlation of total PA levels between the baseline and 5-year surveys. All P values are <0.0001.

^‡^The decline of men's total PA was significantly larger than that of women's, as tested by the Student’s t-test (P=0.0345).

Table S5. Odds ratios (ORs) for sarcopenia according to quartiles of non-leisure-time PA during sitting and standing/walking

Quartiles of non-leisure-time PA (MET-hr/day) P for trend

Q1 Q2 Q3 Q4

Sitting (light PA) as a predictor

Men

Number of cases 41 5 39 52

Number of controls 675 100 866 1148

Unadjusted OR (95% CI) 1 (Ref) 0.82 (0.32-2.13) 0.74 (0.47-1.16) 0.75 (0.49-1.14) 0.1576

Age-adjusted OR (95% CI) 1 (Ref) 0.88 (0.33-2.32) 0.79 (0.50-1.24) 0.87 (0.57-1.34) 0.4757

Multivariable-adjusted OR^*^ (95% CI) 1 (Ref) 0.78 (0.29-2.12) 0.84 (0.52-1.36) 0.97 (0.61-1.53) 0.8561

Women

Number of cases 24 39 3 61

Number of controls 649 1075 45 1678

Unadjusted OR (95% CI) 1 (Ref) 0.98 (0.59-1.65) 1.80 (0.52-6.22) 0.98 (0.61-1.59) 0.9777

Age-adjusted OR (95% CI) 1 (Ref) 1.03 (0.61-1.74) 1.70 (0.48-5.98) 1.10 (0.68-1.79) 0.6557

Multivariable-adjusted OR^*^ (95% CI) 1 (Ref) 0.95 (0.56-1.63) 1.63 (0.44-5.95) 1.01 (0.61-1.67) 0.8549

Standing or walking (moderate PA) as a predictor

Men

Number of cases 21 25 51 40

Number of controls 415 677 933 764

Unadjusted OR (95% CI) 1 (Ref) 0.73 (0.40-1.32) 1.08 (0.64-1.82) 1.04 (0.60-1.78) 0.4378

Age-adjusted OR (95% CI) 1 (Ref) 0.76 (0.42-1.39) 1.03 (0.60-1.74) 1.19 (0.68-2.07) 0.2463

Multivariable-adjusted OR^*^ (95% CI) 1 (Ref) 0.85 (0.46-1.58) 1.14 (0.66-1.99) 1.25 (0.70-2.25) 0.2378

Women

Number of cases 37 29 26 35

Number of controls 674 854 910 1009

Unadjusted OR (95% CI) 1 (Ref) 0.62 (0.38-1.02) 0.52 (0.31-0.87) 0.63 (0.39-1.01) 0.0576

Age-adjusted OR (95% CI) 1 (Ref) 0.64 (0.39-1.06) 0.51 (0.30-0.86) 0.74 (0.46-1.19) 0.1668

Multivariable-adjusted OR^*^ (95% CI) 1 (Ref) 0.68 (0.41-1.14) 0.54 (0.32-0.91) 0.79 (0.48-1.30) 0.2461

*Note*. Cutoff values are 2.2, 2.6, and 5.2 for men, and 2.6, 4.9, and 5.2 for women for light PA; 5.5, 10.0, and 18.0 for men, and 9.5, 14.0, and 20.0 for women for moderate PA.

^*^Adjusted for age, marital status, education, occupation, BMI at baseline survey, energy, smoking, drinking, and history of stroke and diabetes.

Table S6. Odds ratios (ORs) for sarcopenia according to four levels of leisure-time physical activity (PA)

Levels of leisure-time PA (MET-hr/day) P for trend

Tertiles for scores >0

Zero Low Medium High

Men

Number of cases 45 24 30 38

Number of controls 907 614 628 640

Unadjusted OR (95% CI) 1 (ref) 0.79 (0.48-1.31) 0.96 (0.60-1.55) 1.20 (0.77-1.87) 0.3952

Age-adjusted OR (95% CI) 1 (ref) 0.70 (0.42-1.17) 0.68 (0.42-1.11) 0.69 (0.44-1.09) 0.1158

Multivariable-adjusted OR* (95% CI) 1 (ref) 0.85 (0.50-1.44) 0.78 (0.46-1.30) 0.77 (0.47-1.26) 0.2833

Women

Number of cases 28 26 40 33

Number of controls 1002 813 813 819

Unadjusted OR (95% CI) 1 (ref) 1.14 (0.67-1.97) 1.76 (1.08-2.88) 1.44 (0.86-2.41) 0.0605

Age-adjusted OR (95% CI) 1 (ref) 1.10 (0.64-1.91) 1.37 (0.83-2.26) 0.94 (0.56-1.58) 0.9906

Multivariable-adjusted OR^*^ (95% CI) 1 (ref) 1.07 (0.61-1.88) 1.26 (0.75-2.12) 0.89 (0.52-1.52) 0.7729

Men and women combined

Number of cases 73 50 70 71

Number of controls 1909 1427 1441 1459

Unadjusted OR (95% CI) 1 (ref) 0.92 (0.64-1.32) 1.27 (0.91-1.78) 1.27 (0.91-1.78) 0.0646

Age-adjusted OR (95% CI) 1 (ref) 0.86 (0.59-1.25) 0.96 (0.68-1.35) 0.79 (0.56-1.11) 0.2510

Multivariable-adjusted OR† (95% CI) 1 (ref) 0.92 (0.63-1.34) 0.99 (0.69-1.41) 0.80 (0.56-1.14) 0.2712

*Note*. Cutoff values are 1.1 and 3.8 for men, and 1.1 and 3.6 for women.

^*^Adjusted for age, marital status, education, occupation, BMI at baseline survey, energy, smoking, drinking, history of stroke and diabetes, and non-leisure-time PA.

^†^Adjusted for sex, age, marital status, education, occupation, BMI at baseline survey, energy, smoking, drinking, history of stroke and diabetes, and non-leisure-time PA.

Table S7. Odds ratios (ORs) for sarcopenia according to levels of leisure-time moderate-to-vigorous PAs

Quartiles of leisure-time moderate-to-vigorous PA (MET-hr/day) P for trend

Tertiles for scores >0

Zero Low Medium High

Men

Number of cases 58 17 22 40

Number of controls 1162 359 601 667

Unadjusted OR (95% CI) 1 (Ref) 0.95 (0.55-1.65) 0.73 (0.45-1.21) 1.20 (0.79-1.82) 0.6832

Age-adjusted OR (95% CI) 1 (Ref) 1.02 (0.58-1.79) 0.60 (0.36-0.99) 0.75 (0.49-1.15) 0.0826

Multivariable-adjusted OR^*^ (95% CI) 1 (Ref) 1.15 (0.64-2.07) 0.67 (0.39-1.14) 0.81 (0.51-1.28) 0.2188

Women

Number of cases 40 14 38 35

Number of controls 1277 487 846 837

Unadjusted OR (95% CI) 1 (Ref) 0.92 (0.50-1.70) 1.43 (0.91-2.26) 1.34 (0.84-2.12) 0.1098

Age-adjusted OR (95% CI) 1 (Ref) 0.95 (0.51-1.77) 1.18 (0.75-1.87) 0.92 (0.58-1.47) 0.9284

Multivariable-adjusted OR^*^ (95% CI) 1 (Ref) 0.88 (0.46-1.66) 1.16 (0.72-1.86) 0.85 (0.52-1.39) 0.7276

Men and women combined

Number of cases 98 31 60 75

Number of controls 2439 846 1447 1504

Unadjusted OR (95% CI) 1 (Ref) 0.91 (0.60-1.38) 1.03 (0.74-1.43) 1.24 (0.91-1.69) 0.1879

Age-adjusted OR (95% CI) 1 (Ref) 0.97 (0.64-1.47) 0.85 (0.61-1.19) 0.82 (0.60-1.12) 0.1809

Multivariable-adjusted OR^*^ (95% CI) 1 (Ref) 0.98 (0.64-1.51) 0.89 (0.63-1.26) 0.82 (0.59-1.13) 0.2093

*Note*. Leisure-time moderate-to-vigorous PA included walking quickly and light to moderate exercise. Cutoff values for tertiles of leisure-time light-to-moderate PA are 0.7 and 3.0 for men, and 0.6, and 3.0 for women.

^*^Adjusted for sex, age, marital status, education, occupation, BMI at baseline survey, energy, smoking, drinking, history of stroke and diabetes, and non-leisure-time PA.

Table S8. Odds ratios (ORs) for sarcopenia according to quartiles of changes in total PA during the five years

Quartiles of changes in total PA (MET-hr/day) during the 5 years P for trend

Q1 Q2 Q3 Q4

Men

Number of cases 54 31 24 28

Number of controls 882 634 635 638

Unadjusted OR (95% CI) 1 (ref) 0.80 (0.51-1.26) 0.62 (0.38-1.01) 0.72 (0.45-1.14) 0.0762

Age-adjusted OR (95% CI) 1 (ref) 0.75 (0.47-1.19) 0.61 (0.37-1.00) 0.70 (0.44-1.13) 0.0719

Multivariable-adjusted OR^‡^ (95% CI) 1 (ref) 0.78 (0.48-1.26) 0.68 (0.40-1.14) 0.70 (0.43-1.15) 0.1105

Women

Number of cases 50 27 25 25

Number of controls 1126 769 770 782

Unadjusted OR (95% CI) 1 (ref) 0.79 (0.49-1.27) 0.73 (0.45-1.19) 0.72 (0.44-1.17) 0.1422

Age-adjusted OR (95% CI) 1 (ref) 0.73 (0.45-1.18) 0.72 (0.44-1.18) 0.66 (0.40-1.09) 0.0891

Multivariable-adjusted OR^‡^ (95% CI) 1 (ref) 0.67 (0.41-1.10) 0.65 (0.39-1.08) 0.63 (0.38-1.04) 0.0545

Men and women combined

Number of cases 104 58 49 53

Number of controls 2008 1403 1405 1420

Unadjusted OR (95% CI) 1 (ref) 0.80 (0.58-1.11) 0.67 (0.48-0.95) 0.72 (0.51-1.01) 0.0227

Age-adjusted OR (95% CI) 1 (ref) 0.74 (0.53-1.03) 0.66 (0.47-0.94) 0.68 (0.48-0.96) 0.0123

Multivariable-adjusted OR^§^ (95% CI) 1 (ref) 0.73 (0.52-1.03) 0.66 (0.46-0.95) 0.67 (0.47-0.96) 0.0145

*Note*. Cutoff values for total PA at the 5-year survey are 35.2, 40.9, and 50.8 for men and 34.9, 38.8, and 45.8 for women, and those for changes in total PA during the 5 years are −6.5, −0.3, and 4.5 for men and −4.6, −0.3, and 4.2 for women.

^*^Adjusted for age, marital status, education, occupation, BMI at baseline survey, energy, smoking, drinking, and history of stroke and diabetes at baseline

^†^Adjusted for sex, age, marital status, education, occupation, BMI at baseline survey, energy, smoking, drinking, and history of stroke and diabetes at baseline

^‡^Adjusted for total PA at baseline, age, marital status, education, occupation, BMI at baseline survey, energy, smoking, drinking, and history of stroke and diabetes at baseline

^§^Adjusted for total PA at baseline, sex, age, marital status, education, occupation, BMI at baseline survey, energy, smoking, drinking, and history of stroke and diabetes at baseline

Table S9. Differences in percent frequencies of age, sex, and total physical activity (PA) quartiles at baseline between the subsample and the total sample of the two cohorts.

Subsample Total sample Difference in

(N = 6,971) (N = 45,071) percent frequency^*^

Age (years)

40 - 49 444 (6.4%) 9,064 (20.1%) –13.7%

50 - 59 1,538 (22.1%) 12,972 (28.8%) –6.7%

60 - 69 3,822 (54.8%) 16,632 (36.9%) 17.9%

70 - 74 1,167 (16.7%) 6,403 (14.2%) 2.5%

Sex

Men 3,177 (45.6%) 21,880 (48.5%) –3.0%

Women 3,794 (54.4%) 23,191 (51.5%) 3.0%

Quartiles of total PA (MET-h/day)

Q1 (<35.0) 1,403 (20.1%) 11,177 (24.8%) –4.7%

Q2 (35.0-40.2) 1,879 (27.0%) 11,337 (25.2%) 1.8%

Q3 (40.3-49.8) 1,958 (28.1%) 11,286 (25.0%) 3.0%

Q4 (≥49.9) 1,731 (24.8%) 11,271 (25.0%) –0.2%

^*^Percent frequency of the total sample subtracted from percent frequency of the subsample.
